# Advanced therapeutic strategy for radiation-induced osteosarcoma in the skull base: a case report and review

**DOI:** 10.1186/1748-717X-7-136

**Published:** 2012-08-10

**Authors:** Shoko Merrit Yamada, Yudo Ishii, So Yamada, Shigehiko Kuribayashi, Shinichiro Kumita, Akira Matsuno

**Affiliations:** 1 Department of Neurosurgery, Teikyo University Chiba Medical Center, 3426-3 Anesaki, Ichihara, Chiba, 299-0111, Japan; 2 Department of Neurosurgery, Nippon Medical School, 1-1-5 Sendagi, Bunkyo-ku, Tokyo, 113-8603, Japan; 3 Department of Radiology, Nippon Medical School, 1-1-5 Sendagi, Bunkyo-ku, Tokyo, 113-8603, Japan

**Keywords:** Osteosarcoma, Skull base, Radiation-induced, Surgery, Chemotherapy, Cyber-knife

## Abstract

A review of patients with skull base osteosarcoma secondary to radiation (radiation-induced osteosarcoma: RIOS) of the pituitary tumor shows the mean survival of approximately 7 months (2 weeks – 16 months). This warning prognosis seems to stem from two factors, 1) the anatomical complexity of the skull base for total resection of the tumor, and 2) standard adjuvant therapies for the tumor yet to be established. Contrary to the general belief, the authors report an unusually long survival of a 75-year-old woman with a history of osteosarcoma that developed in the same sequence 20 years after pituitary tumor radiation. On her recent admission, she complained of frontal headaches and MRI studies showed a tumor in the sphenoid sinus. Endoscopic trans-nasal tumor removal allowed for histological diagnosis of an osteosarcoma. However, further rapid tumor growth necessitated a radical tumor resection followed by a combined chemotherapy with ifosfamide, cisplatin, and etoposide (ICE). Despite temporary suppression of the tumor growth, the chemotherapy was discontinued due to severe pancytopenia that occurred after three courses of treatment. Shortly after the discontinuation of ICE therapy, the tumor size increased again rapidly, requiring a novel radiation therapy, Cyber-knife treatment. Following this radiation, the tumor growth was arrested, and the patient remains healthy without neurological symptoms over 24 months. The outcome of Cyber-knife in this case suggests that this specific therapy must be considered for the unresectable skull base RIOS.

## Background

The incidence of radiation-induced osteosarcoma (RIOS) is from 0.01% to 0.03% of all irradiated patients [1,2], and accounts for 5.5% of all osteosarcomas [2]. Histologically, RIOS is characterized by more aggressive features than primary osteosarcoma [3,4]. Particularly in craniofacial osteosarcomas, the 5-year survival rate is 70% in primary osteosarcomas and 17% in RIOS [5,6]. This discrepancy in prognosis reflects the difficulty in total resection of the latter, because of its anatomical features. As a contrast, we present a case of skull base RIOS in a patient with much longer survival than any reported cases.

## Case presentation

**Figure 1:**
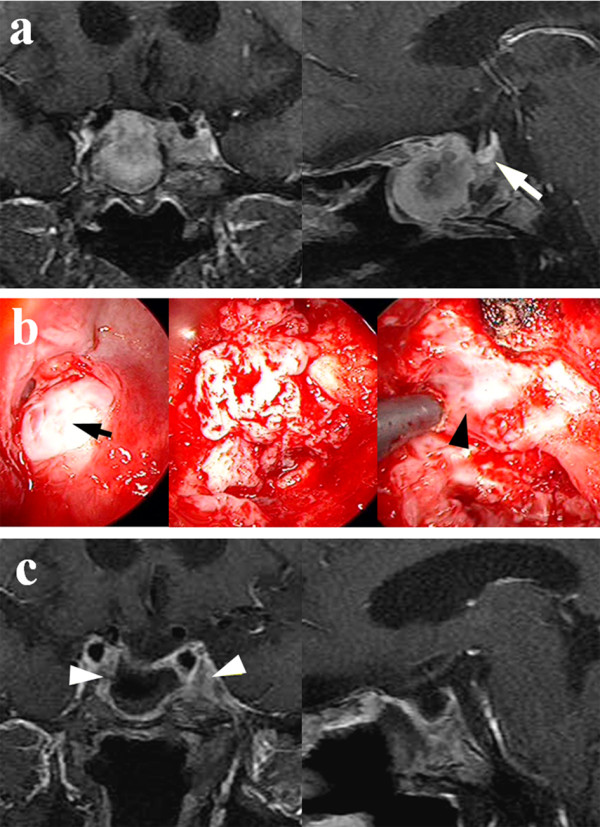
**Before and after the first tumor resection. A.** MRI shows an enhanced mass in the sphenoid sinus. The tumor extends into both cavernous sinuses surrounding internal carotid arteries. The pituitary gland and stalk are clearly identified (white arrow), and there is no tumor invasion in the sellaturcica. **B.** The photo shows a trans-nasal endoscopic view of the tumor. The anterior wall of sphenoid sinus is absent, and a whitish tumor (black arrow) is seen through the nasal cavity. The tumor was elastic and bled easily. There is no tumor invasion in the dura mater in the sellaturcica (black arrowhead). **C.** The tumor in the sphenoid sinus was totally removed; however, residual tumor is noted in cavernous sinuses (white arrowheads).

**Figure 2:**
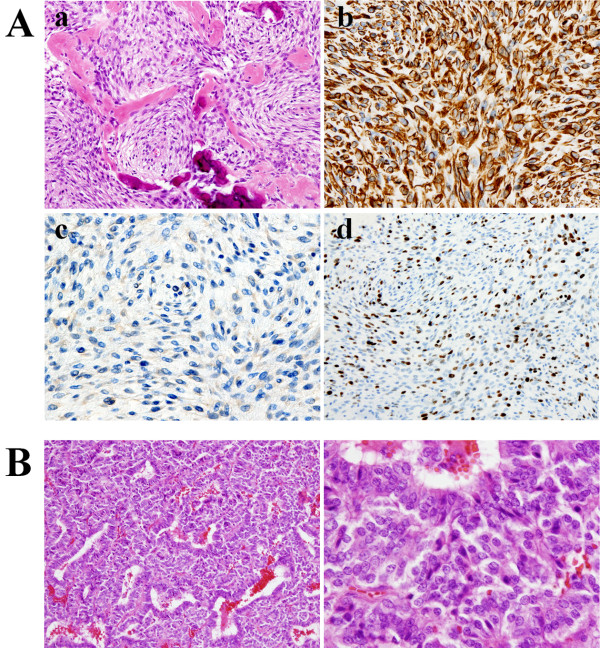
**Histological findings. A: a**. Hematoxylin and eosin staining shows condensed tumor cells with pleomorphism, and depositions of osseous tissue are recognized. **b**. Vimentin was strongly positive. **c.** Anti-cytokeratin (CAM 5.2) staining is negative, indicative of no malignancy of squamous epithelium. **d**. Ki-67 scoring (MIB-1 index) is extremely high at 20%. **B:** The tumor tissue, resected from the patient 20 years ago, consists of typical pituitary adenoma cells, which are entirely different from the pathological features identified in A.

In November 2009, a 75-year-old Asian woman presented with a chief complaint of severe frontal headaches for several weeks. The history dated back to 20 years earlier when she underwent subtotal resection of a nonfunctioning pituitary adenoma, followed by irradiation (50 Gy). Magnetic resonance imaging (MRI) showed an enhanced mass that filled the entire sphenoid sinus cavity, farther invading both cavernous sinuses without involvement of the pituitary gland and stalk (Figure 1A white arrow). Subtotal resection of a whitish, elastic, and easily bleeding tumor was performed by trans-nasal endoscopic approach (Figure 1B black arrow). There was no tumor invasion seen in the dura through the defective floor of the sella (Figure 1B black arrowhead). Postoperatively, a residual tumor was noted in both cavernous sinuses (Figure 1C white arrowheads). The patient was free of headaches after surgery and was discharged to home in December 2009. The tumor was diagnosed histologically as osteosarcoma (Figure 2A), with high cellularity and pleomorphism, mixed with osteoid osseous component (Figure 2A-a). Vimentin was strongly positive (Figure 2A-b), while anti-cytokeratin (CAM 5.2) was negative (Figure 2A-c). Ki-67 scoring (MIB-1 index) was extremely high at 20% (Figure 2A-d), corresponding to the diagnosis. These findings are clearly different from those of the previously resected pituitary adenoma (Figure 2B).

**Figure 3:**
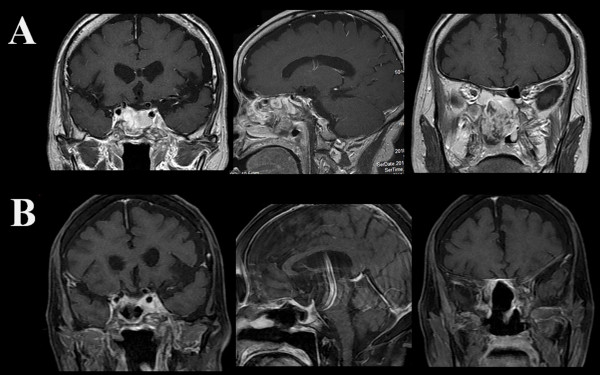
**Recurrence of the tumor. A**. Four months after the first surgery, MRI demonstrates rapid regrowth of the tumor filling sphenoid and ethmoid sinuses and nasal cavity. **B**. A residual tumor is seen in the cavernous sinuses after massive tumor resection.

In March 2010, the patient returned to clinic, complaining of stuffy nose. Follow-up MRI demonstrated extensive regrowth of the tumor in the nasal cavity (Figure 3A). In April, a second subtotal trans-nasal endoscopic tumor resection was performed without extending to the cavernous sinus portion (Figure 3B). Several courses of chemotherapy started immediately with ifosfamide, cisplatin, and etoposide (ICE) at 2-month intervals, but was discontinued one month after the third course due to severe pancytopenia (white blood cell 400/mm^3^, platelet <10,000/mm^3^) associated with malaise. Although a few doses of granulocyte-colony stimulating factor (GCSF: Lenograstim) effectively ameliorated mild pancytopenia after the first and second chemotherapy, the severe pancytopenia developed after the third chemotherapy, and was not adequately controlled by several administrations of GCSF.

**Figure 4:**
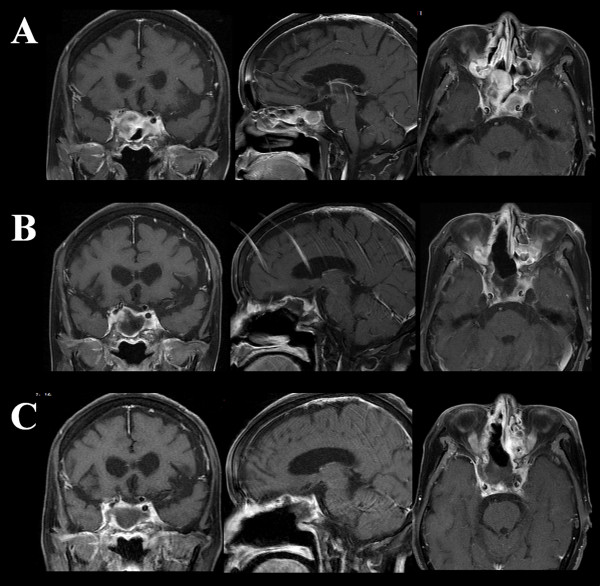
**After chemotherapy and cyber-knife treatment. A**. Tumor regrowth is noted after discontinuation of ICE chemotherapy on MRI. **B**. The cavernous sinus portion of the tumor is noted after the third tumor resection. **C**. No obvious tumor recurrence is identified seven months after cyber-knife radiation.

In January 2011, she was noted to have right visual impairment with optic atrophy, only light perception preserved. MRI showed tumor extension into the ethmoid and sphenoid sinuses, and the right optic canal (Figure 4A). In February, the third subtotal trans-nasal endoscopic tumor removal was performed, leaving the portion attached to the walls of ethmoid and sphenoid sinuses, and in the cavernous sinus intact (Figure 4B). In the end of the month, Cyber-knife radiation was delivered to the residual tumor in five fractions. Target volume was 11716 mm^3^, collimator was 20 mm in diameter, marginal dose was 39.38 Gy, and side dose was 55.71 Gy. In September 2011, MRI showed no farther tumor regrowth in the entire area of radiation (Figure 4C, Table 1). She lives at home and continues to be asymptomatic for 24 months since the first surgery.

**Table 1:** Review of skull base radiation-induced osteosarcoma

**Authors (year)**	**Age (year) Sex**	**Primary tumor**	**Radiation dose (Gy)**	**Latency (years)**	**Site of OS**	**Treatment**	**Survival period**
Amine & Sugar (1976) [13]	16 F	pituitary adenoma	51.0	10	sella	radiation	5 weeks
Tanaka et al. (1989) [14]	57 M	craniopharyngioma	110.0	15	sphenoid wing	embolization surgery	2 weeks
Salvati et al. (1994) [15]	45 M	pituitary adenoma	44.0	12	sphenoid	radiation (50 Gy)	16 months
Gnanalingham et al. (2002) [8]	67 F	pituitary adenoma	52.2	14	sella	surgery	-
Hansen et al. (2003) [16]	29 M	pituitary adenoma	52.0	22	sella–clivus	surgery	(short)
Bembo et al. (2004) [17]	45 M	pituitary adenoma	46.8	5	sella	surgery	7 weeks
Patel et al. (2011) [18]	44 F	craniopharyngioma	60.0	9	sphenoid–ethmoid sinus	chemotherapy	16 months
Present case	75 F	pituitary adenoma	50.0	20	sphenoid sinus	surgery chemotherapy cyber-knife	24 months ~

## Discussion and conclusions

In 1948, Cahan et al. described the following conditions as a definition of the radiation-induced sarcoma: 1) the initial and secondary neoplasms are of significantly different histological type; 2) the secondary neoplasm must arise within the irradiated area; 3) there must be a long latency period after radiation (>5 years); and 4) all sarcomas must be proven histologically [7]. Our case fulfilled all these four conditions, and the diagnosis of RIOS was established. The most frequent radiation-induced sarcoma is fibrosarcoma, and osteosarcoma is extremely rare [8,9]. Based on our review of seven reports, the median survival time of skull base RIOS is 7 months (range 2 weeks to 16 months). The main causes of the early death after surgery were bleeding from the tumor or internal carotid artery (ICA), and occlusion of ICA. Fatal ICA damage can be attributed to aggressive tumor removal in the cavernous sinus. Although some reports described the effectiveness of chemotherapy using methotrexate, ifosfamide, doxorubicin, carboplatin, vincristine, or etoposide, their results are variable [10-12], and a definite protocol has not been established. In the presence of uncontrollable rapid regrowth of the intracranial RIOS, which are known to be resistant to any type of treatment, we selected Cyber-knife irradiation as a novel treatment. This radiation method has three advantages over others: 1) it utilizes its ability to be focused on the target, using the multi-angled planes. Therefore, the dose delivery is superior to Linear accelerator (Linac), which provides the radiation energy only in the single plane, 2) the sizes of tumors of irregular shapes can be evaluated by CT or MRI during Cyber-knife treatment, and radiation doses can be adjusted to maximize the radiation effects. Since the tumor of our patient extended into the cavernous sinus, and different air sinuses, three dimensional evaluation can be done during treatment, 3) Cyber-knife does not require cumbersome equipment as proton beam and gamma knife treatment. In the literature, no report on Cyber-knife treatment is found to demonstrate its effectiveness on RIOS. Our patient’s remarkable daily activity speaks itself for the control of RIOS regrowth by Cyber-knife. We present a case of skull base RIOS with an exceptionally long survival by a combination of endoscopic resection and cyber-knife radiation.

## Consent

Written informed consent was obtained from the patient for publication of this Case report and any accompanying images. A copy of the written consent is available for review by the Editor-in-Chief of this journal.

## Abbreviations

RIOS: Radiation-induced osteosarcoma; ICE: Ifosfamide, cisplatin, and etoposide; MRI: Magnetic resonance imaging.

## Competing interest

The authors declare that they have no competing interests.

## Authors’ contributions

Guarantor of the integrity of the study: SMY. Study concepts: SMY and AM. Study design: SMY. Definition of intellectual content: SK and SK. Literature research: SMY and SY. Clinical studies: YI and SY. Data acquisition: SMY. Data analysis: SMY, YI, and AM. Statistical analysis: SK and SK. Manuscript preparation: SMY. Manuscript review: AM. All authors read and approved the final manuscript. Manuscript English language editing: JAM Post, Inc.

## Source of funding

The authors have no funding declared.
